# Cow-pox and Measles at the Same Time, in the Same Patient

**Published:** 1837

**Authors:** 


### Art. VIII.—Cow-pox and Measles at the same time, in the same patient.

Eighteen months ago, the following case occurred in our practice. A female infant, 11 months old, was vaccinated on the 20th of February; on the 24th, there was a healthy and well formed vesicle, which, however, was rather large for the 5th day. On the following night the child became feverish, thirsty and restless, and the next morning had a palpable morbillous efflorescence, which, by night, was general over its body, with a cough, hoarseness and watery eyes. On the 26th, no areola had yet begun to form around the vesicle, and it had scarcely grown the least from the fifth day. On the 28th, the efflorescence disappeared, but the vesicle remained unchanged. On the 29th, it was larger, and a slight areola had begun to appear. The child's health was restored. On the first of March, the vesicle was still larger, but the areola had not increased, and no appearance of scabbing existed. Circumstances prevented our seeing the patient again. The measles were more or less prevalent in the city at the time, but the child was not known to have been exposed to the contagion. The reader will note, that, in this case, the eruptive fever lasted but a short time before the efflorescence came out, that it was transient, and that the catarrhal symptoms followed it; still we presume the disease to have been measles, modified by the action of the vaccine virus; and that the two contagions exerted on each other, as to their effects, a reciprocal influence. D.

